# Dr. Charles W. M. Brown

**Published:** 1911-12

**Authors:** 


Dr. Charles W. M. Brown died at Elmira, October 29, 1911. He was graduated from the Harvard Medical School, 1876. He had been a vice-president of the Medical Society of the State of New York.

